# Tropomyosins: Potential Biomarkers for Urothelial Bladder Cancer

**DOI:** 10.3390/ijms20051102

**Published:** 2019-03-04

**Authors:** Nada Humayun-Zakaria, Roland Arnold, Anshita Goel, Douglas Ward, Stuart Savill, Richard T. Bryan

**Affiliations:** 1 Institute of Cancer and Genomic Sciences, College of Medicine and Dental Sciences, University of Birmingham, Birmingham B15 2TT, UK; R.Arnold.2@bham.ac.uk (R.A.); A.Goel@bham.ac.uk (A.G.); D.G.WARD@bham.ac.uk (D.W.); R.T.Bryan@bham.ac.uk (R.T.B.); 2 North Wales Clinical Research Centre, Betsi Cadwaladr University Health Board, Wrexham LL13 7YP, UK; STUART.SAVILL@wales.nhs.uk

**Keywords:** tropomyosin, TPM, urothelial bladder cancer, NMIBC

## Abstract

Despite the incidence and prevalence of urothelial bladder cancer (UBC), few advances in treatment and diagnosis have been made in recent years. In this review, we discuss potential biomarker candidates: the tropomyosin family of genes, encoded by four loci in the human genome. The expression of these genes is tissue-specific. Tropomyosins are responsible for diverse cellular roles, most notably based upon their interplay with actin to maintain cellular processes, integrity and structure. Tropomyosins exhibit a large variety of splice forms, and altered isoform expression levels have been associated with cancer, including UBC. Notably, tropomyosin isoforms are detectable in urine, offering the potential for non-invasive diagnosis and risk-stratification. This review collates the basic knowledge on tropomyosin and its isoforms, and discusses their relationships with cancer-related phenomena, most specifically in UBC.

## 1. Introduction

Each year, circa 100,000 patients in the UK undergo investigation for haematuria, the most common symptom of urothelial bladder cancer (UBC). Around 10% of these patients are subsequently diagnosed with UBC [1], of which 75–80% will have non-muscle-invasive disease (NMIBC: stages Ta/T1/Tis) [2]. The remaining 20–25% of patients are diagnosed with muscle-invasive disease (MIBC: stages T2+) following transurethral resection of bladder tumour (TURBT), and undergo further staging investigations before receiving definitive treatment.

For NMIBC patients, long-term cystoscopic surveillance is the mainstay of management following initial transurethral resection (TURBT) and adjuvant intravesical therapy [3]. Cystoscopy is expensive, invasive, uncomfortable, and carries morbidity [4]. Consequently, bladder cancer is one of the most expensive malignancies to manage on a per-patient basis from diagnosis to death, the majority of which is attributable to the long-term treatment and surveillance of NMIBC [5]. Furthermore, significant patient burden and healthcare costs are incurred by the investigation of patients with haematuria, over 80% of whom are subsequently diagnosed with non-malignant conditions or no abnormality [1].

The development of accurate urinary biomarkers for the non-invasive detection of UBC could transform patient pathways by reducing reliance on cystoscopy, which is burdensome for patients and expensive for healthcare providers [6]. Hence, non-invasive diagnosis is a key unmet need for UBC [7], and has become a global research challenge. A number of commercial biomarkers exist and, to date, six have been approved by the FDA for the detection and surveillance of UBC (NMP22, NMP22 BladderChek Test, BTA TRAK, BTA Stat, UroVysion^®^ FISH and ImmunoCyt). However, none have been accepted into routine clinical practice due to poor performance and/or poor evidence [3,8,9]. Notably, patients require tests that are at least as accurate as cystoscopy [10]. However, cystoscopy itself is not infallible: sensitivity has been estimated at 85%, with 87% specificity [6,11] (and it is highly operator-dependent). Thus, as a viable alternative to cystoscopy for patients and healthcare providers alike, non-invasive urinary biomarkers need to match or exceed the sensitivity and specificity of conventional flexible cystoscopy, ideally with utility to also risk-stratify patients above and beyond conventional clinico-pathological tools [3]. This review focuses on the potential use of tropomyosin (TPM) in the detection and stratification of bladder cancer. TPM performs isoform-specific functions within normal cells of the urothelium, and alteration in the isoform expression and concomitant spliceform switching can be observed in the event of bladder carcinogenesis, as will be discussed later in Section 4.

## 2. Tropomyosin

### 2.1. Background and Diversity

Tropomyosin was first identified as a myofibrillar structural protein, involved in contractile function within skeletal and cardiac muscle [12]. TPM is a key regulator in the excitation–contraction coupling mechanism within muscle cells that allows synchronous movements of the myosin heads with the corresponding actin filaments. Though tropomyosins are largely seen to be part of muscle cells, their functions are not limited to muscle cells alone. TPMs have been found in all living cells, including yeast, with the exception of plants and bacteria [13]. TPMs form coiled-coil actin-binding dimers involved in the structural and functional integrity of both the thin-filaments in muscle and the actin cytoskeleton in non-muscle cells. They form an integral component in the regulation of cellular viability and differentiation, and any alterations in gene expression impact cell morphogenesis [13]. The tropomyosin genes thus influence cellular motility, cell–cell adhesion and extracellular interactions, as seen in their contribution to cancer cell metastasis via cell proliferation, migration and invasion [13]. As we see in Section 5 and Section 8, isoform usage and expression levels change during carcinogenesis in tissue-specific patterns, highlighting the complexity of TPM gene and splicing regulation.

The biodiversity of the gene products is achieved by the expression of multiple isoforms and was revealed in greater detail following the publication of the complete human genome [14]. The human genome contains only four genes of the tropomyosin family, however, by the use of differential promoter sequences and splicing events, more than 40 different tropomyosin isoforms are generated from these four genes. The four loci are named α-, β-, γ- and δ- genes (formally known as *TPM1*, *TPM2*, *TPM3* and *TPM4*, respectively). The gene loci are widely dispersed and are not interlinked: 15q22, 9p13, 1q22 and 19p13 [15]. Historically, research on the usage of tropomyosin isoforms for tissue-specific functions generated a myriad of names that were used interchangeably. To mitigate the resulting naming confusion, an agreed nomenclature has been devised and approved by the National Centre for Biotechnology Information (NCBI) to identify the particular isoform in question [16]. For instance, the *TPM1* gene encodes for the Tm2 isoform, hence named as Tpm1.2. For the purpose of clarity, this article will use the approved latest version of tropomyosin nomenclature (Table 1).

TPM isoforms are commonly divided into two categories on the basis of their molecular weight: high-molecular weight (HMW) tropomyosins are ~284 amino acids in length (32–40 kDa) and low-molecular weight (LMW) TPMs are ~248 amino acids in length (28–33 kDa) [17]. Of the four TPM genes, three give rise to both LMW and HMW isoforms (*TPM1*, *3* and *4*), whereas *TPM2* encodes only HMW isoforms [17]. An overview of common isoforms and their molecular weight classification can be found in Table 1. All TPM genes have an initial transcriptional promoter at exon 1a. The LMW TPMs result from alternative promoter usage, with the possibility of using either exon 1a or an internal promoter found in an intron upstream of exon 1b [17]. The numbering of the exons is possible because the members of the gene family share sequence similarity at the individual exon level. Variable expression levels of LMW isoforms result from the ability of the genes to incorporate exon 6a internal splice variants: promoters at exon 9a, 9b, 9c and 9d from *TPM1*; exon 9a and 9b from *TPM3*; and exon 9a from TPM4 [17].

### 2.2. Function

Within non-muscle cells, actin forms an arbitrary pattern of intracellular cytoskeleton bound to structural proteins that play an important role in multiple cellular processes as well as in the maintenance of cell structure and integrity. TPMs are involved in the regulation of actin’s interaction with binding proteins, as well as in the stabilization of the actin filament and its assembly kinetics [18,19,20]. Stable polymerized actin filaments are also referred to as *stress fibres*. The complexity of tropomyosin isoforms points to numerous tissue-specific and cell-specific functions. TPMs regulate actin polymerization through their protective ability, decreasing the rate by which actin is removed from the pointed end of the filament, which is prone to depolymerisation [20]. The relative binding capabilities of TPMs with actin are inversely proportional to their molecular weight. HMW isoforms have a 3-fold increased propensity to co-localize with actin filaments (as seen in skeletal and cardiac muscle) compared to LMW isoforms. Conversely, LMW TPMs are present in peripheral ruffles, stress fibres, or diffuse dot locations within the cell [18]. For example, Tpm3.1 has a strong affinity for actin that prevents Arp2/3-dependent branching and severing of stable actin polymers by proteins such as ADF/cofilin [20]. Disruption of the stress fibres can impact cell morphogenesis, motility and stability—phenomena observed in early tumourigenesis.

Conversely, cofilin in abundance may act synergistically with specific TPM isoforms and increase actin dynamics in normal and transformed cells [21]. Gelsolin is also known to compete with TPM in binding to actin through depolymerisation, severing and branching of actin filaments that can lead to formation of lamellipodia. This activity is regulated by isoform-specific TPMs that are associated with the target filament [22]. It can be deduced that TPMs limit lamellipodia formation and cellular migration/invasion. Myosin-motor activity is regulated by TPM in an isoform-specific manner. Fascin is another actin-bundling protein that works synergistically with Tpm1.7, and is found in abundance in filopodia [23]. It is feasible that the actin-filament-binding abilities of TPMs relative to their molecular weights could provide a route to the discovery of prognostic biomarkers.

### 2.3. Splice Form Variants

Although over 40 different TPM isoforms have been identified to date, it should be borne in mind how many of these isoforms actually give rise to functional protein molecules. According to the latest gene annotation information available (Ensembl Release 94, GRCh38) supported by RNA-sequencing data, the gene products are:

1. *TPM1*—19 protein-coding isoforms and 6 nonsense-mediated decay (NMD) candidates
2. *TPM2*—8 protein-coding isoforms
3. *TPM3*—12 protein-coding and 2 NMD candidates
4. *TPM4*—14 protein-coding and 5 NMD candidates

Amongst the protein-coding isoforms, the amount of supporting evidence varies (supported by expressed sequence tags or RNA-sequencing data) and their expression is postulated to be tissue-specific [24]. In addition to the tissue-level heterogeneity of TPM isoforms, sub-cellular localization is also utilized to compartmentalize cellular roles. A large number of studies have reported mechanisms by which TPM genes generate isoform diversity [13]: for example, *TPM1* codes Tpm1.6 and Tpm1.7 (Tm2 and Tm3, respectively), yet also produces differential isoforms of Tpm1.8 and Tpm1.9 (Tm5a and Tm5b) via alternative splicing events. These events are tissue-specific and have distinct functions in their destined locations [25].

## 3. TPM Expression and Signalling Pathways in Cancer

**Figure 1:**
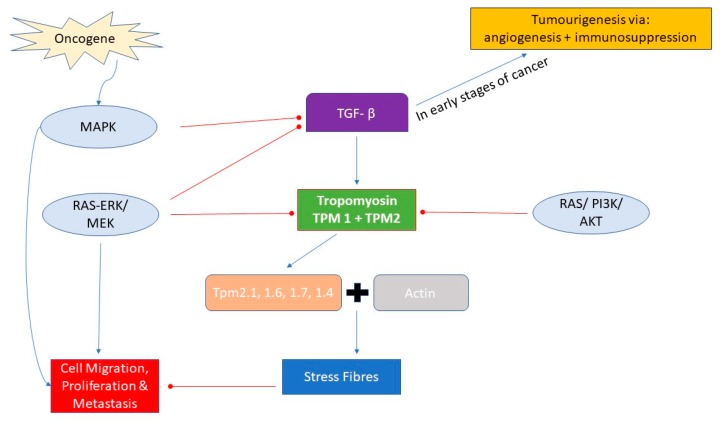
Cellular pathways involved in regulation of TPM binding with actin to form stress fibres to stabilize cellular structure and prevent cell motility.

The interactions of tropomyosin isoforms with their actin-binding proteins influences the functional fate of individual cells, maintained by the signalling pathways responsible for apoptosis, and hence the regulation of cell cycle and prevention of tumour growth in vivo. Alterations of TPM expression are a key feature of the phenotypic changes of transformed cells. Oncogene-mediated alteration within signalling pathways affects TPM expression levels. Mutagenic alterations mediated by Smad and MAPK pathways play a pivotal role in development of carcinogenesis in UBC [26]. The transforming growth factor beta (TGF-β) family of cytokines play important roles as tumour suppressors by inducing pro-apoptotic pathways in normal epithelial cells [27]. In the early stages of tumorigenesis in most epithelial cells, alterations within TGF-β regulation in transformed cells cause a shift towards the progression of disease, as a result of immunosuppression and pro-angiogenesis [28]. TGF-β interacts with TPM by upregulating the expression of *TPM1* and *TPM2*, encoding for the HMW isoforms Tpm2.1, 1.6, 1.7 and 1.4 (Tm1, 2, 3 and 6 respectively). These isoforms are necessary for the formation of stress fibres, reducing cell motility and migration. Activation of the Ras-ERK pathway inhibits the TGF-β induction of stress fibres by suppressing the expression of tropomyosin, leading to a more motile and invasive phenotype [26]. Additional pathways identified in the propagation of UBC include members of the p53/Rb and RTK/RAS/P13K pathway. Notably, genetic alterations within Notch and Hedgehog signalling pathways are responsible for tumour progression [29]. These cellular pathways either directly or indirectly inhibit TPM gene expression, thereby decreasing the formation of TPM–actin polymers, destabilizing cellular stability, and promoting motility and spread (Figure 1).

## 4. Tropomyosin and Urothelial Bladder Cancer

### 4.1. Potential Molecular Mechanisms of Regulation of TPM Isoform Usage

In 2000, Hanahan and Weinberg described the six “hallmarks of cancer” that we have previously described in the context of UBC carcinogenesis [30,31,32]. A revised version in 2011 added two more important mechanisms—“reprogramming of energy metabolism” and “evading immune destruction”—that sustain tumour progression and disease [33]. UBC tumourigenesis is complex and multifactorial, and the specific molecular processes are beyond the scope of this review. Hence, we will focus on the contribution and effects of TPMs within these processes.

Normal urothelial cells contain an abundance of HMW TPM isoforms of the *TPM1* and *TPM2* genes [34]. These (Tpm1.6 and Tpm1.7) are incorporated within actin bundles to stabilize stress fibre formation, maintain cellular integrity and prevent motility [20]. In the event of DNA damage, *TPM1* rescues transformed cells by acting in conjunction with long non-coding RNA MEG3 (Maternally expressed gene 3) in inhibiting cell proliferation, causing cell cycle arrest in stage G0/G1 (with dampening of G2/M), and promoting apoptosis (with upregulation of Bcl2-associated X (BaX) and caspase-3, and downregulation of Bcl2 and cyclin D1) [35].

Cell–cell adhesion is maintained and monitored in the epithelial zonula adherens. Cell adhesion and cytokinesis are established by Tpm2.1, and are involved in the process of anoikis (a form of cell death induced by detachment from the extracellular matrix) [20]. Tpm2.1 expression is similar in both NMIBC and MIBC. It has been hypothesised that the expression of *TPM2* is an early event during bladder carcinogenesis [34,36]: in the initiation phase of tumorigenesis in UBC, HMW isoforms Tpm1.6 and Tpm2.1 have markedly reduced expression levels [34]. With ongoing transformation incited by either oncogenes, carcinogens, oncogenic DNA and/or RNA viruses, immunity to antigrowth signals is achieved. A notable association exists between *TPM1*, miR-96 (microRNA 96), and the long non-coding RNA MEG3 (Maternally expressed gene 3), where *MEG3* and *TPM1* are downregulated and miR-96 levels are upregulated. Regulation of isoform-specific expression by micro-RNA is generated by targeting the FOXQ1 gene, which induces epithelial–mesenchymal transition (EMT, the process by which the epithelial cells acquire characteristics of mesenchymal cells), promoting the progression of disease [35]. Suppression of *TPM1* is a hallmark of EMT: loss of polarity and disassembly of cell junctions as a precursor to invasion and metastasis.

In UBC, the MAPK pathway promotes limitless replicative capability, resulting in uncontrolled cellular proliferation, growth and invasion [31]. MAPK activation induces TPM isoform switching due to alternative promotor usage and maintains the ability of transformed cells to express high levels of Tpm3.1 [35]. Tpm3.1 is an important TPM isoform associated with multiple cancers, including UBC, and is involved in a multitude of cellular processes. Tpm3.1 regulates the uptake of glucose within the adipose cells via the insulin-stimulated GLUT4 transporter in the plasma membrane [37], generation of stress fibres, excitation–contraction coupling, and MEK/ERK-mediated regulation of cell proliferation [20]. Tpm3.1 also results in the failure of MyoIb (unconventional myosin Ib, a key intracellular organelle transporter) to not recognize actin filaments, destabilizing the cell’s structural integrity [38]. Furthermore, degradation of matrix and invasion into the extracellular matrix (ECM) is propagated by the formation of lamellipodia, resulting from the overexpression of LMW isoforms [35,39]. These transformed cells eventually invade and escape from the ECM to metastasize to distant sites. Through the ectopic expression of isoforms Tpm1.6 and Tpm2.1 in cancerous cells, sustainability is maintained in a simulated normal-like morphology, which inhibits anchorage-independent growth (Table 2) [34].

As outlined above, in conjunction with other members of the cytoskeletal machinery, TPMs can mediate differential and alternative cellular capabilities for malignant transformation and invasion into (and escape from) the ECM. Furthermore, due to their many isoforms and tissue- and compartment-specific regulation, TPMs have the potential to support malignant transformation simply by aberrant regulation. This aberrant regulation may be mediated through alternative transcription factor binding site usage, as indicated by the aforementioned work in a non-cancerous cell-line by Savill et al. [17].

### 4.2. Isoform Switching Due to Alternative Promoter Usage

Using the UCSC Genome Browser and the ENCODE regulatory tracks (histone marks and transcription factor binding sites), we performed an initial assessment of potential mechanisms for tropomyosin dysregulation by alternative transcriptional activation. These data are preliminary, since the underlying datasets are incomplete (the cell lines, the epigenetic marks, and transcription factors available have been chosen by the ENCODE consortium selectively, and do not represent all cell and tissue types). Thus, our observations should be evaluated further in the context of the cell-line and tissue type, and ultimately integrated with data from UBC. However, with these caveats, an initial inspection of the TPM gene family isoforms results in the following observations:

- *TPM1* has differential presence of RXRA (retinoid X receptor alpha) transcription factor binding-site (TFBS) for its LMW isoforms, as observed in two cell lines: H1-hESC (a human embryonic stem cell line) and HepG2 (a human liver cancer cell line).
- *TPM3* and *TPM4* have differential presence of the E2F1 TFBS for its LMW isoforms, as observed in the HeLa (cervical cancer) and MCF-7 (a breast cancer cell line) cell lines.

RXRA has been implicated in UBC [40] and E2F1 has been reported as an independent risk factor for UBC prognosis [41]. Given the differential presence of established cancer-related TFBS in alternative TPM promoters, and previous observations in different cancer settings, expression differences in TPM genes should be studied at the isoform level.

## 5. TPM as a Marker for Cancer Prognosis

### 5.1. UBC

HMW isoforms of TPMs bind tightly to actin polymers, stabilizing cellular structure and building a network of stress fibres to facilitate anchorage-dependent growth. The reduction of these isoforms and replacement by LMW isoforms—as evidenced in some malignant cells—promotes cell mobility and metastatic spread. Given their functional implications in cancer and cancer progression, TPMs—and especially TPM isoform expression—may serve as future biomarkers for disease state and prognosis. For example, decreased *TPM1* expression is seen more often in high-grade urothelial carcinoma, MIBC and metastatic bladder cancer than in low-grade urothelial carcinoma, NMIBC and non-metastatic bladder cancer [35]. Low expression of *TPM1* is related to worse grade and stage, as well as metastasis [35].

### 5.2. Other Cancers

Elevated levels of *TPM3* expression have been found related to very poor prognosis in glioma [42]. In hepatocellular carcinoma (HCC) cell lines, amplification of *TPM3* leads to upregulation of Snail-mediated EMT and downregulation of E-cadherin which is ultimately responsible for the migration and invasion of HCC and worse prognosis [43]. In other forms of urinary cancer, viz. renal cell carcinoma, diminished levels of *TPM1* have been found to have strong prognostic associations—deemed a tumour-suppressor gene in this solid tumour, its association with tumour size, Fuhrman grade and smoking status has been well established, with an inversely proportional relationship between *TPM1* expression and prognosis [44].

## 6. Tropomyosins as Gene Fusion Partners

Gene fusion events constitute a class of transcriptional events in which one part of a gene is fused to a fragment of another, and are observed in many different cancer types [45]. Such fusion events may be mediated by chromosomal translocations, or may be created at the transcriptomic level by potential trans-splicing [46] or transcriptional read-through [47]. Although many such fusions may be passenger events, several fusion events have been found to be recurring driving events in cancer. A prominent example is the EWS-FLI1 fusion in Ewing sarcoma, which is identified in more than 90% of cases and acts as a tumour-inducing aberrant transcription factor [48]. For *TPM3* and *TPM4*, recurrent fusion events have been found in a variety of cancers. One of the most frequently-occurring *TPM* fusion events is fusion to the ALK (anaplastic lymphoma kinase) gene [47,49,50]. *ALK* itself is a promiscuous fusion partner. Although this tyrosine kinase appears to have its normal activity in neuronal development, it may hijack 5′ elements from other genes such as *TPM3*, allowing for altered (activated) expression and the ability to self-associate, leading to a consecutive activated kinase domain from the 3′ end of ALK (reviewed in [47]). *TPM3* and *TPM4* have been found to be frequently fused to *ALK* fusions (with 25 (*TPM3*) and 6 (*TPM4*) instances, respectively, listed in the curated set of fusions in the COSMIC database [51]). In a study of kinase fusion events in the TCGA cohort, Stransky et al. [50] also detected one *TPM1–ALK* fusion event in the 250 bladder cancer samples investigated. Another tyrosine kinase found fused with *TPM3* is ROS1 (proto-oncogene tyrosine-protein kinase), found in lung cancer [52] and spitzoid melanomas [53], resulting in a similar change of kinase function as for ALK. NTRK1 (neurotrophic receptor tyrosine kinase 1) also fuses to *TPM3*; seen in more than 5% of thyroid cancers, the event again connects the *TPM3* 5′ end with the NTRK1 kinase domain. In summary, *TPMs* can be used by kinases as fusion partners to result in high levels of expression. Future studies would be necessary to determine if they also interfere with the normal function of the *TPM* genes and the associated cellular processes.

## 7. Detection in Urine

Due to the evidence that TPM isoforms may be prognostic, they might also serve as biomarkers for disease and disease state. In UBC, it would be especially interesting to investigate if a urinary diagnostic test based on tropomyosins and their isoforms could be feasible. Such a test could be based on the quantification of relative isoform usage, which might be more robust against dilution and admixture of other protein/RNA species. Furthermore, due to their abundance within skeletal and cardiac muscle as noted earlier, it could be hypothesized that the secretion or release of TPM isoforms within urinary exosomes could be indicative of breach of the basement membrane and invasion into and beyond the muscular layer of the bladder wall. Note that microRNA-96 has been reported as a potential biomarker, detectable in urine [54], which might be a proxy to TPM isoform usage. In the deep RNA sequencing study of Sin et al. [55], comparing healthy and bladder cancer patients by differential gene expression in order to detect biomarkers, tropomyosins were not reported to be significantly differentially expressed between the healthy and the disease state. However, this simply indicates that the total TPM level stayed similar (differential isoform usage was not investigated). Zhao et al. created a comprehensive catalogue of proteins detectable by different proteomic approaches from the pooled urine of 24 healthy volunteers, and detected *TPM1*, *TPM2* and *TPM4* [56]. In the same work, the authors also compiled a database including five recent comprehensive proteomic studies of urine, in which *TPM3* was detected twice, and *TPM1* and *TPM4* once (excluding their own studies and vesicle-based studies). Existing evidence shows that TPMs can be detected by mass spectrometry in urine, but does not provide information on splice form variants, and thus this aspect of biomarker utility remains uninvestigated.

In addition, RNA sequencing and proteomic research has also been conducted on urinary exosomes. Using Vesiclepedia [57], we evaluated whether TPMs are, in principle, detectable in urine samples in such experiments. We found *TPM1* and *TPM3* in two experiments and *TPM4* in three out of a total 76 human urine experiments, all detected by mass spectrometry. This indicates some recurrence of TPMs in urinary exosomes. It also illustrates regular inclusion of TPMs in the exosomes in contrast to most detected genes, which occurred in only one study and might therefore represent a one-off occurrence.

**Figure 2:**
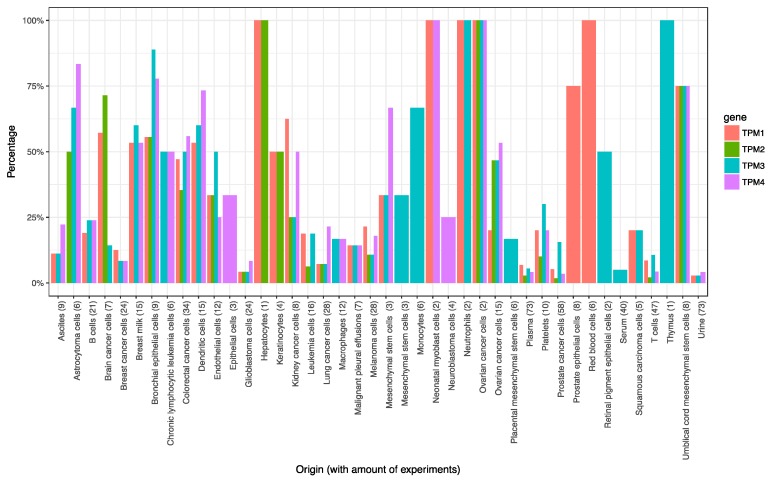
Frequency of *TPM1*, *2*, *3* and *4* in experiments reported in Vesiclepedia. *X*-axis: targeted substrate/origin of the study including the amount of experiments in brackets; *Y*-axis: frequency in experiments (human-based data only, substrates with no *TPM* detected not shown).

Aside from urine, the frequency of TPMs is much higher and detectable in cell exosomes and other biofluids (Figure 2). We conducted the same analysis for microRNA-96 (using the section of Vesiclepedia for microRNA-targeted experiments) and found hsa_mir96-5p to be more specific to urine, where it is observed frequently (5 out of 7 experiments). Even though not all the experiments which report either *TPM*s or mir96 are in a UBC setting (only one experiment in UBC urine is reported in Vesiclepedia), the reported evidence indicates, in principle, the detectability in urinary exosomes by mass spectrometry and microRNA sequencing, respectively. The shortcomings encountered with shotgun proteomics may be mitigated in the future by using affinity-capture to enrich for TPMs, the development of specific bioinformatics approaches to identify specific isoforms and ultimately the use of targeted mass spectrometry (MRM: multiple reaction monitoring) for isoform-specific peptides.

## 8. Tropomyosin Isoforms in Other Cancers

Although this review focuses on TPM in UBC, we also report findings from other cancers, highlighting the general importance of tropomyosins in cancer.

### 8.1. Malignant Breast Epithelial Cells

HMW tropomyosin isoforms of *TPM1* and *TPM2* are expressed in normal breast epithelial cells along with the expression of LMW isoforms of *TPM1* and *TPM4*. It has been observed that the expression of these genes is downregulated in some (although not all) malignant breast cells. An additional four novel isoforms of the *TPM1* gene (that encode TPM isoforms) have been identified in nine malignant breast epithelial cell lines and are not otherwise expressed in normal adult breast tissue [58]. The *TPM2* isoform has been found markedly upregulated in aggressive forms of breast cancer with lymph node metastases [58,59], and could potentially serve as a prognostic biomarker.

### 8.2. Cholangiocarcinoma

It has been demonstrated that levels of *TPM1* are downregulated in intrahepatic cholangiocarcinoma and upregulated in extrahepatic cholangiocarcinoma. *TPM1* regulation in this case is regulated by two important cellular pathways (i.e., RAS/PI3K/AKT and RAS/MEK/ERK), and via inhibition of epigenetic mechanisms [60]. These pathways also seem to overlap with UBC and could be targets for future therapies.

### 8.3. Prostate Cancer

Alternative splicing events give rise to several isoforms in normal cells and may not be directly involved in carcinogenesis. However, some isoforms do stimulate cellular expansion and the growth of malignant cells, providing a “tumour microenvironment”. This phenomenon is observed in prostate cancer where mutagenic pathways cause overexpression of the *TPM1* isoform, providing a platform for distant metastases [61].

### 8.4. Colon Cancer

The T84 Colon cancer cell line has been used to isolate a novel isoform of Tpm3.12, named TC22, which has been associated with colonic carcinogenesis. Its presence in benign polyps and increased expression levels by up to 75% in adenomatous dysplastic cells demonstrates a relationship with colon cancer [62].

### 8.5. Oesophageal Cancer

Ras effector regulatory pathways MEK/ERK and PI3K/AKT are compromised in oesophageal squamous cell carcinoma cell lines, leading to downregulation and decreased expression of *TPM1* and *TPM2* [63]. A similar pattern is observed in UBC, where downregulation of HMW TPM isoforms leads to higher grade and stage and poorer prognosis [35].

## 9. Discussion and Conclusions

Whilst tropomyosin isoforms have been investigated for decades, their true potential as cancer biomarkers has not been evaluated to date. Given their complexity of isoforms, as well as tissue- and cell-specific functions, many findings remain to be discovered. They play an integral role in combination with the actin cytoskeleton in the maintenance of cell morphology, and alterations in gene expression may mediate EMT, leading to more aggressive disease and worse survival. With ongoing technological advances, we suggest that the investigation of tropomyosin isoforms could mediate further advances in UBC diagnosis and risk stratification. With the limited knowledge currently available, the detection of novel isoforms remains a challenge. Whether tropomyosin isoforms could be quantified as protein molecules within urine or genetic alterations within alternative splicing events of *TPM* mRNA remains to be uncovered. However, with the availability of genomic data and experimental validation, we are currently investigating the precise role of this enigmatic protein in the pathogenesis of UBC.

## Figures and Tables

**Table 1:** Distribution of molecular weight over well-characterized tropomyosin (TPM) isoforms and genes. HMW: high molecular weight; LMW: low molecular weight.

Tropomyosin Gene	*TPM* Isoforms Common (Old) Name	*TPM* Nomenclature	Size (HMW/LMW)	Molecular Weight (kDa)
***TPM1***	Tm2 Tm3 Tm5a Tm5b Tm6	Tpm 1.6 Tpm 1.7 Tpm 1.8 Tpm 1.9 Tpm 1.4	HMW HMW LMW LMW HMW	36 34 30 30 40
***TPM2***	Tm1	Tpm 2.1	HMW	38
***TPM3***	Tm5NM1	Tpm 3.1	LMW	30
***TPM4***	Tm4.1 Tm4	Tpm 4.1 Tpm 4.2	HMW LMW	30

**Table 2:** TPM isoform usage in urothelial bladder carcinogenesis. EMT: epithelial–mesenchymal transition.

Bladder Cancer Stage	Hallmark	Process	TPM Involvement
**Normal**	Self-sufficiency in growth signals	Normal expression levels of *TPM* genes	Tpm 1.6 and Tpm 1.7—formation of stress fibres, cell stability, reduced motility
**Suppression**	Insensitivity to growth inhibitory signals	Cell-cycle arrest in G0/G1 if proliferating too much *TPM1* + MEG3 causes apoptosis by upregulation of Bcl2 and caspase3	*TPM1*—acts as tumour suppressor
**Initiation**	Evasion of programmed cell death	Decreased expression of *TPM1* and *TPM2* *TPM1* is downregulated due to increased micro-RNA 96 (suppression lost)	Loss of *TPM1*—EMT Tpm 2.1—responsible for anoikis, maintains cell–cell adhesion
**Non-invasive**	Limitless replicative potential	Mutagenic MAPK signalling activated	Tpm3.1—cellular growth, proliferation and motility
**Invasive**	Sustained angiogenesis	Invasion into extracellular matrix (ECM) Angiogenesis stimulated by concurrent pathways	LMW TPMs—formation of lamellipodia, increased cell motility and morphogenesis
**Metastasis**	Tissue invasion and death	Breakthrough ECM with distant spread	LMW isoforms of *TPM3* and *TPM4*—Cancer cell survival, focal adhesion, MEK/ERK-mediated proliferation
	Evading immune destruction	Ectopic expression of *TPM1* and *TPM2* Continued uninhibited anchorage-independent growth	

## Abbreviations

UBC	Urothelial Bladder Cancer
NMIBC	Non-Muscle Invasive Bladder Cancer
MIBC	Muscle Invasive Bladder Cancer
TURBT	Transurethral Resection of Bladder Tumour
TPM	Tropomyosin (protein)
*TPM*	Tropomyosin gene
MAPK	Mitogen Activated Protein Kinase
